# Isothermal Analysis of ThermoFluor Data can readily provide Quantitative Binding Affinities

**DOI:** 10.1038/s41598-018-37072-x

**Published:** 2019-02-25

**Authors:** Nan Bai, Heinrich Roder, Alex Dickson, John Karanicolas

**Affiliations:** 1 Program in Molecular Therapeutics, Fox Chase Cancer Center, Philadelphia, PA 19111 USA; 2 Department of Molecular Biosciences, University of Kansas, Lawrence, KS 66045 USA; 3 Department of Biochemistry & Molecular Biology and Department of Computational Mathematics, Science and Engineering, Michigan State University, East Lansing, MI 48824 USA

## Abstract

Differential scanning fluorimetry (DSF), also known as ThermoFluor or Thermal Shift Assay, has become a commonly-used approach for detecting protein-ligand interactions, particularly in the context of fragment screening. Upon binding to a folded protein, most ligands stabilize the protein; thus, observing an increase in the temperature at which the protein unfolds as a function of ligand concentration can serve as evidence of a direct interaction. While experimental protocols for this assay are well-developed, it is not straightforward to extract binding constants from the resulting data. Because of this, DSF is often used to probe for an interaction, but not to quantify the corresponding binding constant (K_d_). Here, we propose a new approach for analyzing DSF data. Using unfolding curves at varying ligand concentrations, our “isothermal” approach collects from these the fraction of protein that is folded at a single temperature (chosen to be temperature near the unfolding transition). This greatly simplifies the subsequent analysis, because it circumvents the complicating temperature dependence of the binding constant; the resulting constant-temperature system can then be described as a pair of coupled equilibria (protein folding/unfolding and ligand binding/unbinding). The temperature at which the binding constants are determined can also be tuned, by adding chemical denaturants that shift the protein unfolding temperature. We demonstrate the application of this isothermal analysis using experimental data for maltose binding protein binding to maltose, and for two carbonic anhydrase isoforms binding to each of four inhibitors. To facilitate adoption of this new approach, we provide a free and easy-to-use Python program that analyzes thermal unfolding data and implements the isothermal approach described herein (https://sourceforge.net/projects/dsf-fitting).

## Introduction

Differential scanning fluorimetry (DSF), also known as ThermoFluor or Thermal Shift Assay, has become an important label-free technique for biophysical ligand screening and protein engineering^1–5^. Briefly, this method makes use of a dye – typically either SYPRO Orange or 1-anilino-8-naphthalenesulfonate (ANS) – that is quenched in an aqueous environment but becomes strongly fluorescent when bound to exposed hydrophobic groups of a protein. By heating one’s protein of interest in the presence of such a dye, the thermal unfolding transition can be monitored spectrophotometrically. Because ligands that interact with proteins typically stabilize the folded protein, this leads to a shift in the midpoint of the unfolding transition (i.e. the melting temperature, T_m_)^6,7^.

The simplicity of this assay makes DSF very straightforward to implement using an RT-PCR thermocycler, it can be inexpensive and fast, and it requires relatively little sample^8^: these advantages have made this approach attractive for screening applications in drug discovery – particularly for moderately-sized fragment libraries^1,2,9^ – and also for protein stability formulation^10,11^. Meanwhile, the fact that this method is label-free and well-suited to detect binding over a wide range of affinities has made DSF one of the most popular approaches in drug discovery for fragment screening^6,12–15^ and for evaluating the “ligandability” of a target protein^16^. While it would be desirable to obtain binding constants at an early stage, for example to prioritize fragment hits on the basis of their ligand efficiency^17^, the magnitudes of the observed T_m_-shifts (at a given ligand concentration) have been shown to correlate only weakly with compounds’ potency measured in other orthogonal assays^18^.

**Figure 1:**
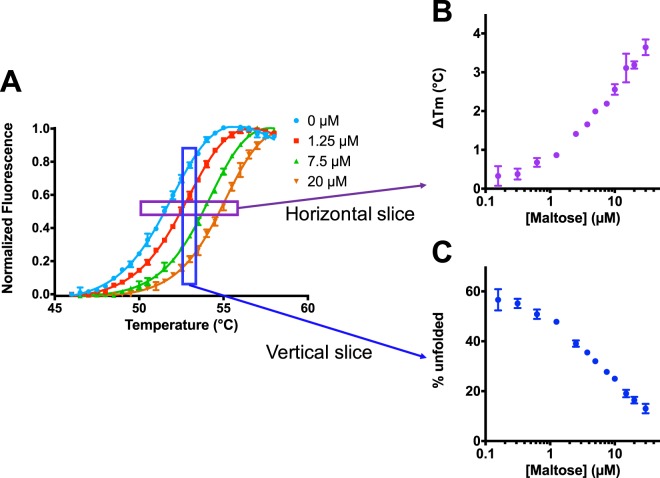
Maltose binding to MBP, as probed via DSF. (**A**) Thermal unfolding of MBP is monitored using SYPRO Orange. Data were collected in the presence of increasing maltose concentrations, leading to a rightward shift in the unfolding transition. (**B**) The T_m_-shift (∆T_m_) is determined by plotting the increase in temperature at which each curve has 50% relative fluorescence, corresponding to a horizontal “slice” of the original data. However, this analysis does not provide the binding affinity of the protein/ligand pair. (**C**) Instead, here we use vertical “slices” of the original data. By plotting – *at a single temperature* – the fraction of protein that is unfolded as a function of ligand concentration (here at 53 °C), the binding affinity can then be easily determined. All data are collected in triplicate, and error bars correspond to the standard error of the mean (some are too small to be seen).

Typical DSF data are shown in Fig. 1A. Here, SYPRO dye is used as a reporter for the extent of unfolding of maltose binding protein (MBP), and the melting temperature from each curve is determined. Using this method, MBP is observed to have a T_m_ of approximately 52.5 °C in the absence of its ligand, maltose. Upon addition of increasing concentrations of maltose, the unfolding transition is shifted to increasingly higher temperatures: this implies that maltose stabilizes MBP, by binding to the natively folded protein.

Dose-response data in DSF experiments are typically presented by showing the T_m_-shift as a function of ligand concentration (Fig. 1B), and there are a number of ways to determine T_m_ from the fluorescence data. One simple method is to take the first derivative of the observed fluorescence data with respect to temperature, and to then identify the maximum value (corresponding to the steepest part of the transition). Other methods instead smoothly fit the whole melting curve, either by using a so-called Boltzmann model^19–22^, or by using a more rigorous “thermodynamic model”^1,3,6,23,24^, or occasionally by using other arbitrary polynomials^25–27^.

The Boltzmann model is the most widely-used approach, in part because it is very user-friendly^19^. The fluorescence at a given temperature is linearly related to the fraction of unfolded protein, which takes the form ${F}_{unfolded}(T)=1+\frac{1}{1+{e}^{\frac{Tm-T}{a}}}$, where *Tm* is the melting temperature and *a* is a parameter that reflects the steepness of the thermal unfolding transition. This model is applied primarily because it provides a sigmoidal shape that can be fit quite well to experimental data, especially when additional fitting parameters are included to account for the fact that the dye itself often has some temperature dependence (Figure S1). Despite its name, however, this equation does not explicitly model the thermodynamic transition^20^: for this reason, the Boltzmann model is not used to garner any information beyond accurately identifying the midpoint of the protein unfolding transition (T_m_)^19,20^, and studies that use this model simply report the presence/absence of binding rather than using this data to determine binding constants^4,19,28–32^.

In studies to date seeking quantitative binding constants, “thermodynamic models” have been used. The simplest of such models write the fraction of unfolded protein as ${F}_{unfolded}(T)=1+\frac{1}{1+{e}^{\frac{{\rm{\Delta }}H(1-T/Tm)-{\rm{\Delta }}{C}_{p}(Tm-T+Tln(T/Tm))}{RT}}}$, where ΔH is the enthalpy change of protein unfolding and ΔCp is the change in heat capacity enthalpy change of protein unfolding (both assumed to be temperature-independent)^3,33,34^. Typically ΔCp is under-determined given the available experimental data, and therefore determined through separate complementary experiments^23^ or estimated from the buried surface area of the folded protein^35,36^, then fixed when fitting the thermal unfolding data. Though more complicated to write down, these models in fact have the same number of the effective free parameters (when ΔCp is fixed at a pre-determined value). Further, these models also have the advantage of using physically meaningful parameters.

Simply determining the T_m_-shift as a function of ligand concentration is not sufficient to provide the binding affinity, however. Although some groups have simply fit these curves using the Hill equation^19,37^ – treating the T_m_ as an arbitrary “observable” that depends on the ligand concentration – this is not a physically reasonable approach. The Hill equation is only applicable when the observable is linearly proportional to the fraction of one of the species that is bound/unbound in solution, and T_m_ is not such a variable. The ∆T_m_ data are also (by definition) drawn from different temperatures: the binding affinity cannot be assumed constant at different temperatures, further making the Hill equation inappropriate for this usage. This point is further underscored by the fact that these experimental data do not correspond to a simple saturation-based ligand titration method^4^: rigorous thermodynamic simulations show that ∆T_m_ should change monotonically with increasing ligand concentration^23,38^, even if this behavior is not always observed in real cases due to artifacts like irreversible protein aggregation^38^.

Instead, correct binding constants have thus far been determined using a more rigorous approach that explicitly considers the temperature-dependent enthalpy, entropy, and heat capacity of both protein folding and ligand binding^3,5^. Using these thermodynamic parameters determined from the complete unfolding transitions, binding constants can subsequently be determined at the T_m_. The means to do so was presented several decades ago^5^, and also in the context of screening for ligands that bind a particular protein^1^. In the earliest cases, these equations were formulated for the weak-binding regime (i.e. high dissociation constants), such that the free ligand concentration can be approximated by the total ligand concentration; these equations have since been extended to avoid the latter assumption^23,38,39^. In all cases, though, the binding constant is determined at the T_m_; together with the binding enthalpy, the van’t Hoff equation can then be used to extrapolate binding constants at other temperatures. Because the binding enthalpy is difficult to determine from the unfolding transition data, this most commonly comes from a knowledge-based estimate^1^ or is measured directly using other techniques like isothermal titration calorimetry (ITC)^40^.

While details of the model have been iteratively improved since the original formulation, the two key elements of the “thermodynamic model” have remained unchanged: a fit of the melting curves is used to obtain multiple thermodynamic parameters, then these are used to calculate the binding constant at T_m_ and potentially (via extrapolation) at other temperatures^3^. These elements of the model also remain the two key practical limitations of DSF. Because of the complexity associated with correctly replicating this analysis, it is often cited in modern studies but not frequently used: DSF is most popular as a qualitative test rather than a quantitative test, with the majority of literature reports reporting T_m_-shifts as shown in Fig. 1B but not attempting to extract binding constants-^8,11,28,29,41–44^. Collectively this has led to a general consensus that the observed T_m_ shifts “cannot be readily transformed into binding affinities”^45^.

Here, we develop and describe a new *isothermal* strategy for analysis of DSF data. Rather than determine the T_m_ values from the raw fluorescence data at each ligand concentration, we instead select a *single* temperature of interest, and *at this temperature* we evaluate the fraction of protein that is folded/unfolded at each ligand concentration (Fig. 1C). Because all of the data used corresponds to the same temperature, no thermodynamic parameters are required; instead, a very simple model of coupled equilibria (protein folding/unfolding and ligand binding/unbinding) describe our system. Furthermore, because we only require the fraction of protein that is unfolded (for a given ligand concentration, at the temperature of interest), the raw data can be fit either with the simple Boltzmann model or with the more rigorous thermodynamic model (Figure S1). Other studies have similarly used isothermal slices of unfolding data, for example in analysis of cellular thermal shift data (CETSA)^46^ and other protein-ligand interactions^47,48^; however, each of these stopped short of using these data to quantitatively determine binding constants. As demonstrated below, here we show that this approach leads to a very simple formulation for determining the binding affinity near the protein’s unfolding temperature, and it provides values consistent with those measured in other orthogonal assays.

## Theory

### Isothermal analysis of ThermoFluor data

DSF experiments, specifically those in which large compound collections are screened, yield melting temperatures that shift either higher or lower when various compounds are added^3,38,39^. Most non-covalent drug-like ligands stabilize their protein target upon selective binding, and accordingly they increase the protein’s T_m_^19,40,49–51^. Conversely, compounds that decrease the protein’s T_m_ are thought to operate by binding the unfolded protein more tightly than the folded protein, by competing with an endogenous (stabilizing) co-factor, or through potentially non-specific effects^15,38,39,52^; some metal ions, like Zn^2+^, can also destabilize proteins^53^. We have excluded from the present analysis cases in which the ligand destabilizes the protein, and we focus solely on the scenario in which the ligand exclusively binds the natively-folded protein with a 1:1 stoichiometry.

Accordingly, we write the protein folding-unfolding reaction as a competitive coupled equilibrium with ligand binding, as follows:

1 $$[U]+[L]\mathop{\,\rightleftharpoons \,}\limits_{{{\rm{K}}}_{{\rm{U}}}}[F]+[L]\mathop{\,\rightleftharpoons \,}\limits_{{{\rm{K}}}_{d}}[FL]$$

where [U] is the concentration of the unfolded protein, [L] is the concentration of free ligand, [F] is the concentration of the folded and unbound protein, and [FL] is the concentration of the protein-ligand complex. K_U_ is the equilibrium constant for the protein unfolding reaction, and K_d_ is the equilibrium constant for the unbinding reaction. Both K_U_ and K_d_ depend on temperature, but both are constant at fixed temperature (and fixed buffer conditions). Intuitively from this scheme, we see that the concentration of unfolded protein goes to zero as the ligand concentration becomes large and drives the equilibrium to the right. Importantly, this scheme assumes each reaction (folding and binding) has no intermediates, and thus can be represented in this two-state manner; we will consider further the implications of this assumption in the Discussion section. We also note that the presence of the reporter dye is not included in our model.

From the conservation of mass and the definitions of these two equilibrium constants, we write the following:

2 $${[P]}_{T}=[F]+[U]+[FL]$$

3 $${[L]}_{T}=[L]+[FL]$$

4 $${K}_{U}=[U]/[F]$$

5 $${K}_{d}=([F]\times [L])/[FL]$$

where [U] is the concentration of the unfolded protein, [L] is the concentration of free ligand, [F] is the concentration of the folded and unbound protein, and [FL] is the concentration of the protein-ligand complex. In Equation 4 we define K_U_ as the equilibrium constant between the *unbound* unfolded and folded states ([U] and [F]). This equilibrium constant is therefore independent of ligand concentration, and reflects the overall fraction of protein that is unfolded/folded *only* when no ligand is present (since inclusion of ligand shifts some of [U] and [F] into the [FL] state). K_d_ is the equilibrium constant for the unbinding reaction. [P]_T_ is the total protein concentration, and [L]_T_ is the total protein concentration (both of which are known). We note that the interaction between the reporter dye and the protein is not explicitly included in this model, though the presence of the dye presumably does contribute to stabilizing the unfolded protein.

Once the raw data have been normalized, fluorescence intensity in the DSF experiment (Fig. 1A) is linearly related to the fraction of the unfolded protein *f*_*u*_. Starting from the definition of *f*_*u*_, we simplify using Equations 2–5 and obtain the following expression:

6 $${f}_{u}=\frac{[U]}{[U]+[F]+[FL]}=\frac{1}{1+((1/{K}_{U})\times (1+[L]/{K}_{d}))}$$

This provides the fraction of unfolded protein in terms of the *free* ligand concentration [L], whereas the known quantity in this experiment is the total ligand concentration [L]_T_. From Equations 2–5 we obtain the following quadratic equation for [L]:

7 $${[L]}^{2}+({[P]}_{T}-{[L]}_{T}+{K}_{d}(1+{K}_{U}))[L]-{[L]}_{T}{K}_{d}(1+{K}_{U})=0$$

Thus, [L] can be written in terms of the *total* ligand concentration [L]_T_ as follows:

8 $$[L]=\frac{1}{2}[({[L]}_{T}-{[P]}_{T}-{K}_{d}(1+{K}_{U}))+\sqrt{{({[P]}_{T}-{[L]}_{T}+{K}_{d}(1+{K}_{U}))}^{2}+4{[L]}_{T}{K}_{d}(1+{K}_{U})}]$$

We note that this expression corresponds to only one root of the quadratic equation, since the other root is unphysical.

Together, Equations 6 and 8 provide a single expression to write *f*_*u*_ in terms of [L]_T_, [P]_T_, K_U_, and K_d_. As expected for the limiting case where [L]_T_ becomes large, we see from this set of equations that *f*_*u*_ goes to zero. Conversely in the limiting case when [L]_T_ goes to zero, we see that [L] goes to zero and thus Equation 6 reduces to the definition of the equilibrium constant for unfolding. Together, these two limits correspond to the endpoints of the data shown in Fig. 1C.

[L]_T_ and [P]_T_ are experimental parameters that are known; our expression for *f*_*u*_ therefore uses only two free parameters (K_U_ and K_d_). These two parameters can be fit to the normalized data at the same time (as we will demonstrate), or alternatively K_U_ can be first determined at the temperature of interest from the thermal unfolding curve in the absence of ligand; this allows fitting of the data in Fig. 1C to be subsequently carried out with a single free parameter (K_d_).

### A simpler approximate solution

Monitoring the fraction of unfolded protein (through dye binding) in this competitive coupled equilibrium (Equation 1) is very much analogous to detecting the fraction of labeled probe molecule in a competitive binding assay. In the latter case, one uses increasing concentrations of the (unlabeled) inhibitor of interest to explore the effect on a (labeled) probe that binds at the same site; the concentrations of all species, as well as the binding affinity of the probe ligand, can then be used to determine the inhibition constant for the unlabeled species from its IC_50_^54^.

Inspired by this analogy, we explored whether the same strategy could be applied here. We summarize our solution for these equations below, and elaborate further in the Appendix (see *Supplemental Materials*).

We again start from Equations 2–5, but this time we solve these equations for the specific scenario in which the total ligand concentration matches the EC_50_. By definition, the EC_50_ is the ligand concentration at which the fraction of unfolded protein is half of that observed in the absence of ligand (note: the EC_50_ is *not* defined by the ligand concentration at which half of the protein is unfolded, since this can happen even before ligand is added, depending on the temperature). For this special case:

9 $${[L]}_{T}={[L]}_{50}+{[FL]}_{50}=E{C}_{50}$$

10 $${[P]}_{T}={[F]}_{50}+{[U]}_{50}+{[FL]}_{50}$$

11 $${K}_{U}={[U]}_{50}/{[F]}_{50}$$

12 $${K}_{d}=({[F]}_{50}\times {[L]}_{50})/{[FL]}_{50}$$

where [U]_50_, [L]_50_, and [F]_50_ are the concentrations of unfolded protein, free ligand, and folded unbound protein at the condition when [L]_T_ = EC_50_. Recall from Equation 4 that K_U_ is defined to be the equilibrium constant between the only the *unbound* unfolded/folded states (not the overall fraction of protein that is unfolded/folded), and thus for this reason Equation 11 does not include any contribution from [FL]_50_.

Correspondingly, in the absence of ligand we write:

13 $${[P]}_{T}={[F]}_{0}+{[U]}_{0}$$

14 $${[L]}_{T}={[L]}_{0}={[FL]}_{0}=0$$

15 $${K}_{U}={[U]}_{0}/{[F]}_{0}$$

From Equations 13–15 we can solve for the fraction unfolded in the absence of ligand (*f*_*u0*_):

16 $${f}_{u0}=\frac{{[U]}_{0}}{{[U]}_{0}+{[F]}_{0}}=\frac{1}{1+1/{K}_{U}}$$

From the definition of EC_50_, we write:

17 $${[U]}_{50}=\frac{{[U]}_{0}}{2}$$

From Equations 15 and 17, we can write [F]_50_ in terms of [U]_0_ and K_U_. Substituting this into Equation 10 yields an expression for [FL]_50_ in terms of [P]_T_, [U]_0_ and K_U_; simplifying this with Equations 15 and 16, we find that at the ligand concentration corresponding to the EC_50_, half of the total protein concentration has ligand bound to it:

18 $${[FL]}_{50}={[P]}_{T}/2$$

This allows solution of Equations 12 and 16 to yield a simple expression for [L]_50_ as well:

19 $${[L]}_{50}=\frac{\,{K}_{d}}{1-{f}_{{\rm{u}}0}}$$

Combining Equations 18 and 19 back into Equation 9, we obtain a simple expression that relates the EC_50_ to K_d_:

20 $$E{C}_{50}=\frac{\,{K}_{d}}{1-{f}_{{\rm{u}}0}}+\frac{{[P]}_{T}}{2}$$

There are no additional assumptions required to reach this equation (e.g. no need to assume that [L] ≈ [L]_T_). This expression is intuitively gratifying, and it highlights the fact that the EC_50_ observed in this experiment cannot be simply interpreted as the K_d_. Most notably, in the limit where ligand binding is very tight (low K_d_), the observed EC_50_ is driven essentially by stoichiometry (enough ligand must be added to match half the number of available sites on the protein); this makes the EC_50_ very insensitive to changes in the K_d_ in this regime, and it suggests that our approach may not be well-suited to determining the binding affinity for very tight interactions. This implication is borne out in real experimental data, as presented at the end of the following section.

Finally, rearranging Equation 20 yields:

21 $${K}_{d}=(1-{f}_{{\rm{u}}0})\times (E{C}_{50}-\frac{{[P]}_{T}}{2})$$

[P]_T_ is a known experimental parameter. *f*_*u0*_ corresponds to the fraction of protein that is unfolded (at the temperature of interest) in the absence of ligand, and thus it can be determined directly from the thermal unfolding curve in the absence of ligand. Even using a very simple and arbitrary fit of *f*_*u0*_ as a function of ligand concentration (e.g. the Hill equation), we can still easily estimate the midpoint of this transition (the ligand’s EC_50_ value): thus, Equation 21 provides a rapid means to estimate the K_d_ when it is undesirable to fit the complete curve using Equations 6 and 8. That said, fitting with the functional form presented in Equations 6 and 8 leads to the most accurate estimate of the midpoint (since the complete curve is used to determine the fitting parameters), and is thus preferred.

## Results

To test the utility of this isothermal fitting approach, we wrote a program in Python that fits the thermal unfolding curves and uses these to solve Equations 6 and 8 presented above. All of the analysis presented below was carried out using this program, and it is freely available for others to use (https://sourceforge.net/projects/dsf-fitting).

### Accuracy and robustness of isothermal analysis

We first sought to test the accuracy of binding affinity values resulting from this isothermal approach. To do so, we generated realistic simulated thermal unfolding curves. The rigorous approach referenced earlier^23,38^ allows the fraction of unfolded protein to be calculated as a function of temperature and ligand concentration, provided thermodynamic parameters that describe protein unfolding in the absence of ligand (ΔH_U_^Tm^, ΔCp_U_^Tm^, T_m_ and K_U_^Tm^) and thermodynamic parameters that describe ligand binding (ΔH_b_^Tm^, ΔCp_b_^Tm^ and K_d_^Tm^). We selected values for each of these parameters by using values for maltose/MBP from the literature where possible, and then assigning reasonable values to the remaining terms such that the resulting curves were qualitatively similar to those observed experimentally for maltose/MBP.

**Figure 2:**
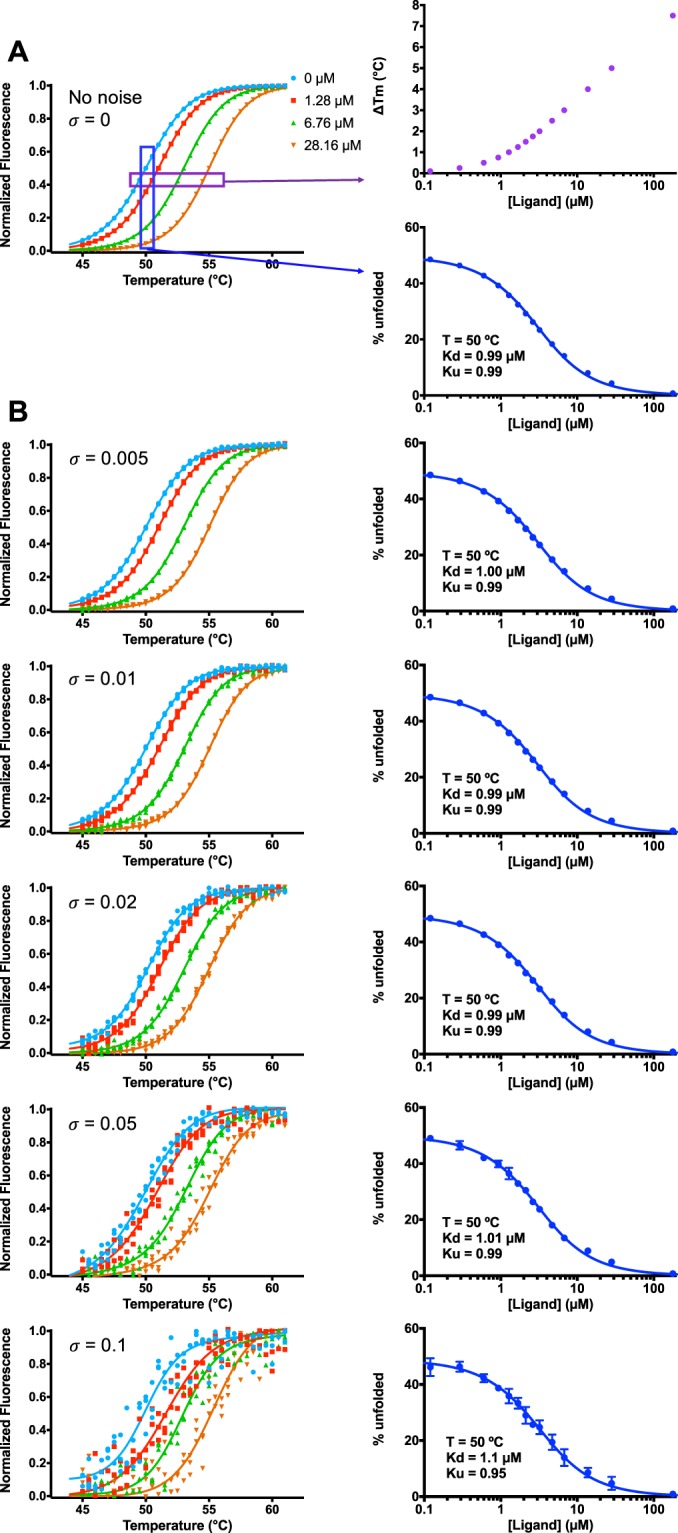
Simulations to explore the consistency and robustness of isothermal analysis. (**A**) Simulated thermal unfolding curves were generated using a thermodynamic model for unfolding and binding. Parameters were set as follows: T_m_ = 50 °C, K_d_^Tm^ = 1 µM, ΔH_U_^Tm^ = 120 kcal mol^−1^, ΔH_b_^Tm^ = −10 kcal mol^−1^, ΔCp_U_^Tm^ = 4 kcal mol^−1^ K^−1^, ΔCp_b_^Tm^ = −0.5 kcal mol^−1^ K^−1^, and total protein concentration = 2 µM. By definition, K_U_^Tm^ = 1. Fitting this simulated data using the simpler isothermal approach yields K_U_^Tm^ = 0.99, and K_d_^Tm^ = 0.99 µM. (**B**) Upon addition of increasing random noise to the simulated unfolding data, the isothermal approach still leads to accurate estimates of K_U_^Tm^ and K_d_^Tm^, up to values exceeding the noise typically present in real experimental data.

In our simulations we set T_m_ in the absence of ligand to be 50 °C, and set the ligand’s dissociation constant (K_d_) to be 1 µM at this temperature. By definition, the unfolding constant (K_U_) at the T_m_ is 1. Using the formulation laid out by others^23,38^ we then generated the corresponding simulated experimental data (Fig. 2A). To make the simulated data suitably approximate the type of experimental data that would be produced in a real experiment, we generated data with temperature intervals and ligand concentrations drawn from the real experimental protocol used earlier (Fig. 1). Reassuringly, analyzing this data using the isothermal approach presented above yields values for both K_d_ and K_U_ that matched those used to generate the simulated data. The previous formulation^23,38^ makes it straightforward to generate unfolding curves from thermodynamic parameters, but the inverse problem is more challenging to solve; by demonstrating that our isothermal methods recovers the underlying K_d_ and K_U_, we show that our method is indeed compatible with the previous formulation of this system.

To explore the robustness of our isothermal analysis, we next introduced noise into the simulated experimental data. Having already normalized each of the simulated unfolding curves to range from 0 to 1, we added to each point a random number drawn from a normal distribution with a given standard deviation (σ). We find that analysis of the resulting data yields K_d_ and K_U_ values that closely match the true value for σ up to 0.05; only once the data becomes noisier than this (σ = 0.1) do the estimates start to differ from those used to generate the unfolding data. The amount of noise in the simulated data at σ = 0.1 is more than observed in typical experiments, suggesting that indeed this isothermal analysis is robust to the random error present in most real experimental data.

### Application to maltose/MBP

**Figure 3:**
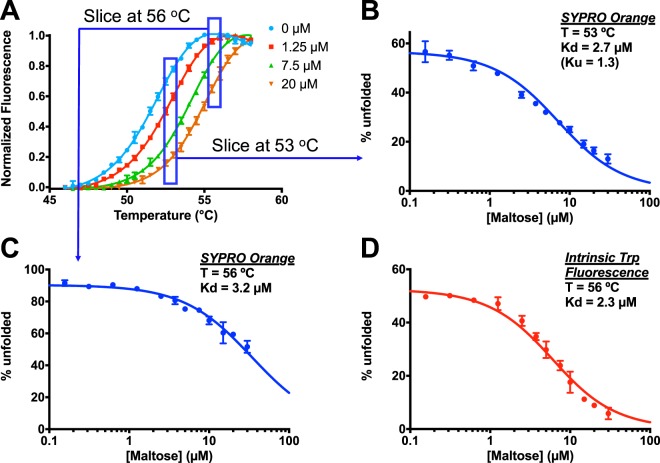
Determination of maltose/MBP binding affinity using isothermal analysis of thermal unfolding data. (**A**) Thermal unfolding of MBP is monitored using SYPRO Orange. Data were collected using 12 increasing maltose concentrations, each in triplicate; 4 representative unfolding curves are shown, after normalization using the Boltzmann equation. (**B**) The fraction of unfolded protein is calculated at 53 °C for each maltose concentration. Fitting using Equations 6 and 8 yields a K_d_ value of 2.7 µM and a K_u_ value of 1.3. (**C**) Extracting instead the fraction of unfolded protein at 56 °C yields a K_d_ value of 3.2 µM. (**D**) The thermal unfolding transition was instead monitored using MBP’s intrinsic tryptophan fluorescence, and the fraction of unfolded protein was calculated at 56 °C for each maltose concentration. Two replicates were carried out for each maltose concentration. Fitting this complementary experimental data using Equations 6 and 8 yields a K_d_ value of 2.3 µM.

As a first test of this approach, we analyzed in further detail the maltose/MBP interaction. This interaction has been frequently-studied using many different forms of calorimetry^35,55^, in part because both the ligand and the protein are soluble to very high concentrations. We returned to the same DSF experimental design described earlier, with 12 increasing concentrations of maltose (Fig. 3A). Given MBP’s T_m_ of about 52.5 °C in the absence of maltose, we first elected to determine the binding affinity for this pair at 53 °C. From individual fits to the complete thermal unfolding curves, we used the thermodynamic model to determine the fraction of unfolded MBP (at 53 °C) at each maltose concentration. We also separately used the Boltzmann model to determine the fraction of unfolded MBP from each thermal unfolding curve, and we found that both methods yielded essentially identical results (Figure S1).

We then used the expressions presented in Equations 6 and 8, to fit the fraction of unfolded MBP at each maltose concentration (Fig. 3B). From the fraction of unfolded protein at each maltose concentration, there are only two free parameters to be fit: the fraction of unfolded MBP in the absence of maltose (K_u_) and the dissociation constant for ligand binding (K_d_). At low maltose concentration, the curve does not go to 100% unfolded, but rather plateaus at about 50% (the first free parameter): this is expected, given that we carried out our analysis at a temperature only slightly above the T_m_. The K_d_ at 53 °C, derived directly from this fit, is 2.7 µM, and the K_U_ value at 53 °C is 1.3. Because we have defined K_U_ as the unfolding constant *in the absence of ligand*, we can also compare the value to that obtained directly from the thermal unfolding curve collected in the absence of ligand: the latter yields a value of 1.5 (at 53 °C), in very close agreement with the value obtained from fitting the binding curve.

We additionally fit these data using the approximate solution shown in Equation 21: given the EC_50_ value of 6.8 µM (estimated by arbitrarily using the Hill equation to fit this curve), this expression yields a K_d_ value of 2.5 µM, in agreement with the more rigorous fit.

One advantage of the isothermal fitting approach is that the binding constants can be directly determined at other temperatures close to the T_m_, provided that there are sufficient differences in the fraction of unfolded protein. As a demonstration of this, we carried out the corresponding analysis using a slightly higher temperature, at 56 °C (Fig. 3C); as expected, the curve from this fit has a higher fraction of unfolded MBP in the absence of maltose. Binding at this slightly elevated temperature yields a very similar K_d_ value of 3.2 µM.

This general approach for extracting dissociation constants is by no means specific to the DSF format; while this is a convenient method for monitoring protein unfolding, the analysis presented here can also be applied to data collected via using other experimental techniques. While DSF is label-free, in principle the presence of SYPRO Orange (or other analogous dyes) may shift the folding equilibrium by preferentially binding to the unfolded state^39^; still, given the analysis outlined above, a systematic shift in protein stability (due to the dye, for example) is not expected to affect the resulting binding affinity. To further explore the effect of the dye, we repeated the experiment described above, this time in the absence of SYPRO Orange and instead relying on MBP’s intrinsic tryptophan fluorescence to monitor unfolding. From an initial experiment in the absence of maltose, we noted that the T_m_ was now about 2.5 °C higher: this confirmed our expectation (and previous reports^39^) that the presence of the dye slightly destabilizes the protein.

Using thermal unfolding traces collected via intrinsic tryptophan fluorescence, we plotted the fraction of unfolded protein at 56 °C, as a function of maltose concentration (Fig. 3D). We again fit these data using the expression from Equation 6 and 8, and again we find that this expression (with two free parameters) appropriately describes the underlying data. The K_d_ value resulting from this fit at 56 °C is 2.3 µM, in close agreement with the value obtained at this temperature using the DSF data. In contrast, the value of K_U_ (in the absence of ligand) at 56 °C is 1.1 using intrinsic tryptophan fluorescence versus 9.4 using SYPRO Orange: this is consistent with the fact that this temperature is very close to the T_m_ determined via intrinsic tryptophan fluorescence but above the T_m_ determined using SYPRO Orange, and again implies that the dye destabilizes the protein.

Using this pair of complementary detection modalities, we have thus confirmed that the general approach laid out above is applicable for analysis of thermal unfolding data, regardless of the experimental means by which the protein’s foldedness is monitored. Inevitably, however, this analysis reports on the binding affinity at a temperature near the protein’s T_m_ (e.g. ±4 °C in this case). In the data presented above we obtain the binding affinity for maltose/MBP at 53 and 56 °C, whereas other techniques to directly probe binding such as ITC and SPR can be used at more physiological temperatures (e.g. room temperature or 37 °C^35,56,57^). In general, extrapolation of binding data from DSF to lower temperatures will require knowledge of the thermodynamic contributions to binding: these may be derived from applying the isothermal approach multiple times over a small temperature range, or from complementary calorimetry experiments^40^/knowledge-based estimates^1^ as described elsewhere. We will test the feasibility of these strategies in future work; here, instead, we next sought to explore whether addition of chaotropic agents would allow us to probe this interaction at lower temperature.

### Using denaturants to access binding constants at lower temperature

Although thermal unfolding may be monitored over a large temperature range, accurate determination of the binding affinity by this method requires that there is a well-resolved range in the fraction of protein that is unfolded; thus, it is natural to carry out this analysis at temperatures close to the T_m_ measured in the absence of ligand. In many cases, however, it is desirable to probe binding at lower, more physiologically-relevant temperatures.

**Figure 4:**
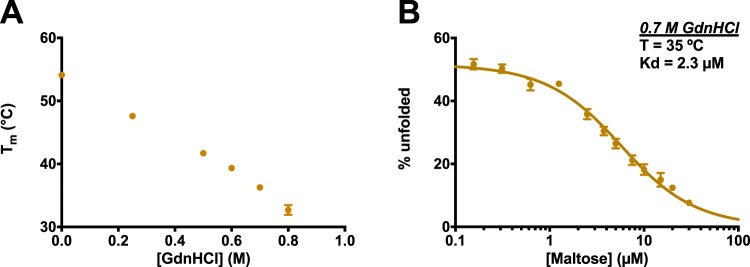
Denaturant effect of MBP unfolding and MBP-maltose binding. (**A**) T_m_ of MBP decreases with increasing GdnHCl concentration. (**B**) MBP-maltose binding with 0.7 M GdnHCl at 35 °C. The value of K_d_ is 2.3 µM. All the experiments were carried out in triplicate. The protein concentration in all the assays was fixed to 2 µM. All assays were taken in the buffer: 120 mM NaCl, 20 mM NaH_2_PO_4_/Na_2_HPO_4_, pH 7.4 with 1% DMSO and the melting program was set to 0.5 °C/min.

To shift MBP’s T_m_ to the desired temperature, we added denaturant to our system. Guanidine hydrochloride (GdnHCl) has been shown not to greatly affect the binding affinities for most protein-ligand interactions, with the exception of strongly ionic ligands^45,58–60^. As a starting point, we used DSF experiments to monitor the MBP’s T_m_ in the presence of increasing denaturant (Fig. 4A); based on these results, we elected to study maltose binding at a GdnHCl concentration of 0.7 M. At this denaturant concentration, we then carried out the same DSF experiments with increasing concentrations of maltose. Having shifted the transition temperature into the physiological range, we now determined the fraction of unfolded MBP at 35 °C (Fig. 4B). Under these conditions, the fit once again appropriately describes the data, and yields a K_d_ value of 2.3 µM: this estimate is consistent with previous studies reporting of values ranging from 0.5 to 2 µM for this interaction^55,56,61^.

By adding this chaotropic agent, then, we have demonstrated that the thermal unfolding transition can be rationally shifted to allow determination of binding affinity at a specific temperature. Importantly, we also observe that – at least in this model system – the presence of GdnHCl does not significantly affect the resulting binding affinity, in agreement with previous reports^45,58–60^.

### Applying this approach to other protein-ligand pairs (without denaturant)

We next applied this approach to study a different protein, with multiple ligands spanning a broad range of binding affinities. We selected another model system that has been frequently used in calorimetric studies^23,24,38,45,62^, carbonic anhydrase (isoforms I and II). From among commercially-available inhibitors of these two enzymes we selected the weak inhibitor sulfanilamide (SULFA, mM K_i_) and the potent inhibitor trifluoromethanesulfonamide (TFMSA, nM K_i_). We also selected two inhibitors with intermediate inhibition constants (µM K_i_), acetazolamide (ACTAZ) and methazolamide (METAZ). The chemical structures of all four inhibitors are shown in Figure S2. There is no chemical denaturant used in these assays.

**Figure 5:**
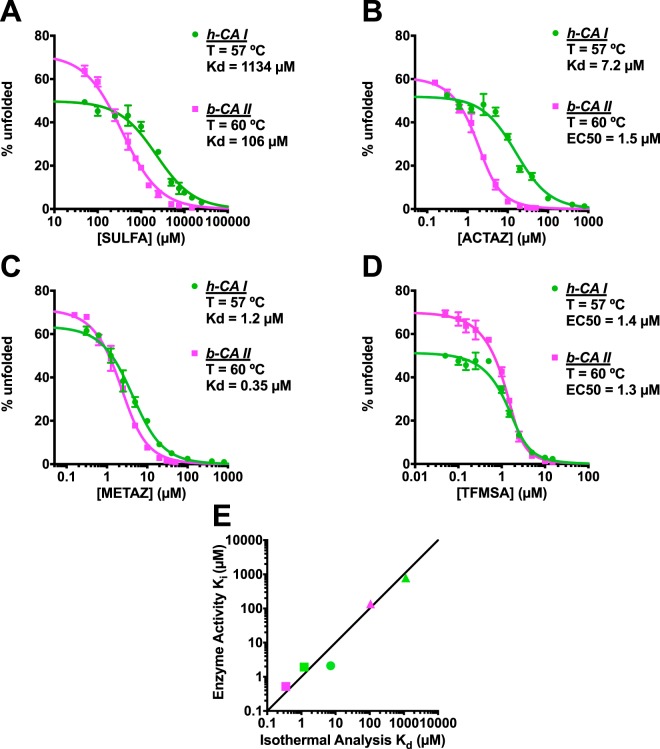
Determination of binding affinities for carbonic anhydrase inhibitors using isothermal analysis of thermal unfolding data. Each inhibitor was characterized with two carbonic anhydrase isoforms, *h*-CA I (*green*) and *b*-CA II (*pink*). (**A**) Analysis of SULFA yielded binding constants of 1.1 mM and 0.1 mM for isoforms *h*-CA I and *b*-CA II. (**B**) ACTAZ gave binding constants of 7.2 µM for *h*-CA I and 1.5 µM as EC_50_ for *b*-CA II. (**C**) METAZ gave binding constants of 1.2 µM and 0.35 µM for the two isoforms. (**D**) TFMSA gave EC_50_ of 1.4 µM and 1.3 µM for the two isoforms. (**E**) Comparison of the binding constants obtained from isothermal analysis of thermal unfolding data versus inhibition constants obtained in an enzyme inhibition activity. The TFMSA/*h*-CA I, TFMSA/*b*-CA II and ACTAZ/*b*-CA II pairs are not included here, because they all have less than 2 µM EC_50_. All experiments were carried out in triplicate.

From thermal unfolding data collected in the absence of inhibitor (not shown), we observed that *b*-CA II was slightly more stable than *h*-CA I. For this reason, we evaluated isothermal binding data for the two isoforms at 60 °C and 57 °C, respectively. From the resulting binding curves (Fig. 5A–D), the relative activities of each inhibitor are clear: SULFA is the weakest, followed by ACTAZ and METAZ, and TFMSA is the most potent. Importantly, this experiment also distinguishes between the two isoforms, with tighter binding observed for *b*-CA II rather than *h*-CA I in all four cases. Overall, this isothermal analysis of the underlying thermal unfolding data provides a range of binding affinities between CA isoforms and their four inhibitors.

As noted earlier with regards to our discussion of Equation 20, the dissociation constant is difficult to obtain from the isothermal analysis of DSF data if K_d_ is much lower than the protein concentration, because binding becomes stoichiometric in this regime. The protein concentration used in these experiments was 2 µM (to allow robust detection of the unfolding transition), and thus Equation 21 shows that this will make it difficult to interpret the K_d_ for EC_50_ values below about 2 µM. For this reason, we used the EC_50_ value to guide interpretation of the results: for cases with EC_50_ values greater than 2 µM we report the K_d_, whereas for cases with EC_50_ less than 2 µM we simply conclude that the K_d_ is less than 0.5 µM (as seen from Equation 20, when [P]_T_ = 2 µM and *f*_*u0*_ is 0.5, a K_d_ value of 0.5 µM will lead to EC_50_ = 2 µM).

To obtain an independent measure of these interactions under identical conditions (temperature and buffer composition), we applied an esterase activity assay and determined inhibition constants (K_i_) for each isoform/inhibitor pair (Table 1). Of the eight protein/ligand pairs, we could not accurately determine the inhibition constant for TFMSA with *h*-CA I due to its potency relative to the enzyme concentration used in our assay: standard Michaelis-Menten analysis cannot be used to determine the inhibition constant for a nanomolar inhibitor at an enzyme concentration of 2 µM. With the exception of this pair, we compared these inhibition constants to the binding constants obtained via DSF: overall there is excellent agreement between the inhibition constants and the binding constants, for activities in the sub-micromolar to millimolar range (Fig. 5E).

**Table 1:** Binding/inhibition constants derived from isothermal analysis of DSF and from enzyme activity assays.

Interaction (ligand/protein)	K_d_ (µM), from DSF	K_i_ (µM), from enzyme assay
SULFA/*h*-CA I	1134 ± 106	786 ± 60
SULFA/*b*-CA II	106 ± 9	136 ± 5
ACTAZ/*h*-CA I	7.2 ± 0.8	2.1 ± 0.2
ACTAZ/*b*-CA II	<0.5	0.48 ± 0.03
METAZ/*h*-CA I	1.2 ± 0.1	1.9 ± 0.2
METAZ/*b*-CA II	0.35 ± 0.03	0.52 ± 0.03
TFMSA/*h*-CA I	<0.5	<1
TFMSA/*b*-CA II	<0.5	0.12 ± 0.04

## Discussion

### Limitations of using thermal unfolding to monitor ligand binding

The simplicity and practical advantages of the DSF format have made this experimental approach very popular, particularly for fragment screening. Nonetheless, there are important considerations that can limit its application with respect to certain ligands and/or proteins.

With respect to ligand screening, certain ligands can naturally interfere with the reporter dye through their own fluorescent properties^1^. In addition, certain ligands may interact with the unfolded protein^3,38^, or promiscuously form non-specific (or covalent) interactions with the protein^63^. Other ligands may also interact with the protein via a stoichiometry other than 1:1, or alternatively, and particularly for small fragment-like compounds, the ligand may interact with a single site on the protein surface using multiple binding modes with comparable affinities^64^. The isothermal formulation we present here does not yet consider any such scenarios, and it is currently restricted to stoichiometric 1:1 binding.

The protein to be studied is also subject to important restrictions. Most importantly, any equilibrium analysis involving protein folding assumes reversibility: this can be difficult to establish conclusively, and many proteins aggregate at elevated temperature. In the course of initiating the studies described here, we explicitly tested whether thermally-unfolded protein could be cooled, and then once again heated to yield the same thermal unfolding transition. In light of well-justified concerns about non-reversible unfolding and aggregation, a number of strategies have been proposed: these include using faster speeds for the unfolding process (to minimize the potential for aggregation)^65,66^ and including in the reaction dyes that explicitly detect protein aggregation^67,68^.

Further, our formulation also makes the important assumption that protein folding/unfolding is two-state, and that there are no substantially-occupied partly-folded intermediates. This is a pervasive assumption, because it greatly simplifies analysis of folding/unfolding data: however, it is also widely-understood that this assumption is not valid in all cases^69^. Relative to traditional thermodynamic characterizations of ligand binding, we expect that the isothermal nature of our analysis will somewhat mitigate the effect of partially-folded states: they will simply be lumped into the folded or unfolded state, depending on their ability to bind ligand. That said, the presence of such states can certainly confound this analysis if they bind the ligand with a different affinity than the folded state (e.g. leading to inadvertent determination of some ensemble-weighted average of the binding constant), or if they lead to errors in calculating the fraction of unfolded protein at the temperature of interest.

### Thermal versus denaturant-induced unfolding

The binding constants derived from this isothermal approach can be accurately determined only in the vicinity of the target protein’s melting temperature, which may not correspond to a temperature of real biological (physiological) interest. We have shown that in such cases denaturant can be used to shift the melting temperature to the desired range; an alternative approach, however, is simply to extract binding constants from the ligand-dependence of the denaturant-induced stability differences.

Indeed, previous studies have laid the groundwork for determining binding constants – at room temperature – based on the denaturation midpoint of protein stability^9,45^. In both of these studies the extent of protein unfolding was monitored by intrinsic tryptophan fluorescence, obviating the need for a reporter dye. That said, an important drawback of this detection modality is the potential for interference from many drug-like compounds, which may limit the range of applicability of this technique. By contrast, typically used reporter dyes such as SYPRO Orange are much less likely to exhibit spectral overlap with potential ligands of interest. Additionally, whereas the data collection for denaturation profiles is usually more rapid, data are typically fit using closely-spaced increments in denaturant concentrations which necessitates more liquid handling to setup the assay.

Overall, we envision that thermal and chemical unfolding can serve as complementary assays, depending on available instrumentation, the importance of obtaining binding constants at a specific temperature, and the spectral nature of the ligands of interest.

### Sensitivity of detecting protein unfolding

As described in the context of Equations 20 and 21, the fraction of protein that is folded depends on the protein concentration relative to the ligand’s K_d_. Under circumstances in which the dissociation constant is much smaller than the protein concentration (i.e. very tight binding), addition of ligand leads to stoichiometric binding and makes it difficult to determine the binding constant. Indeed, we encountered precisely this scenario in our characterization of the carbonic anhydrase inhibitor TFMSA.

The natural solution to this problem is to use very low protein concentration, so that the EC_50_ observed for unfolding is most sensitively dependent on the K_d_ rather than on the protein concentration. This raises a practical consideration, however, because the protein concentration to be used in the assay is determined by the sensitivity with which unfolding can be monitored. The observed fluorescence signal upon dye binding is related to protein size, with larger proteins yielding more signal: for this reason, experiments typically use similar protein concentrations in mass units (i.e. mg/ml) rather than in molar concentrations. For larger proteins, then, lower molar concentrations are accessible for this experiment (provided they unfold in a single cooperative transition), which in turn may allow for characterization of tighter-binding ligands using this approach.

### Thermodynamic models versus our isothermal model

Monitoring protein thermal unfolding transitions is a highly attractive means to access ligand binding, because in principle it can be rapidly setup and deployed for many different protein systems. In addition to DSF/ThermoFluor, analogous data can be collected using other experimental modalities: most notably, probing the protein directly via intrinsic tryptophan fluorescence or circular dichroism (CD) spectroscopy. Regardless of the method by which protein unfolding is monitored, however, the analysis is the same; and indeed, the same thermodynamic models described earlier in the context of DSF have also been applied to thermal unfolding probed via CD^65,70,71^. Unsurprisingly, the challenges associated with applying thermodynamic models to directly quantify ligand binding at different temperatures apply to these other experimental formats as well^72^. Here we have demonstrated that our isothermal analysis can equally well be applied to thermal unfolding transitions monitored via intrinsic tryptophan fluorescence, and we expect this framework to apply equally for data collected using any technique for monitoring protein unfolding.

## Materials and Methods

### Materials

His-tagged maltose-binding protein (MBP) was expressed from a plasmid in *E. coli* and then purified through Ni-chelated Sepharose Fast Flow Resin (GE Healthcare) and HiLoad 16/60 Superdex 75 gel filtration column (GE Healthcare). The protein was exchanged into assay buffer (120 mM NaCl, 20 mM NaH_2_PO_4_/Na_2_HPO_4_, pH 7.4) by dialysis. Both carbonic anhydrases were obtained from a commercial vendor (*h*-CA I (Sigma C4396) and *b*-CA II (Sigma C2522)). All protein concentrations were determined with Quick Start^TM^ Bradford Protein Assay Kit (Bio-Rad, catalog no. 5000201).

Ligands were all obtained from commercial vendors, as follows: maltose (EMD Millipore 105910), acetazolamide (Sigma 97582), methazolamide (Sigma SML0720), sulfanilamide (Sigma 46874), trifluoromethanesulfonamide (Sigma 638455), and 4-nitrophenyl acetate (Sigma N8130).

### Generating simulated unfolding data

Simulated experimental data were generated using the formulation laid out by others^23,38^ that allows the fraction of unfolded protein to be calculated at a given temperature and ligand concentration, provided a set of thermodynamic parameters that describe protein unfolding and ligand binding. Values for these thermodynamic parameters are reported in the caption of Fig. 2. Data were calculated near the T_m_ value in 0.25 °C increments.

### SYPRO DSF assay: experimental protocol

All proteins (MBP, *h*-CA I and *b*-CA II) were used at a final concentration of 2 µM for this assay. SYPRO Orange (Invitrogen S6651) was used at a final concentration of 20X for MBP, and at 10X for the carbonic anhydrases. MBP experiments were carried out in 120 mM NaCl, 20 mM NaH_2_PO_4_/Na_2_HPO_4_, 1% DMSO, pH 7.4. Carbonic anhydrase experiments were carried out in 100 mM NaCl, 20 mM TRIS, 1% DMSO, pH 6.1.

All DSF experiments were carried out with Eppendorf Realplex2 Mastercycler. Each sample was divided to three 50 µL replicates. Sample solutions were dispensed into 96-well optical reaction plate (Thermo Fisher Scientific 4306737) and the plate was sealed with optical PCR plate sheet (Thermo Fisher Scientific AB-1170). Fluorescence intensity was measured via the JOE emission filter (550 nm) and “PTS clear plate” was set as the background for the calibration. Temperature was continuously increased: 0.5 °C/min for MBP, and 1 °C/min for carbonic anhydrase. In the MBP-maltose-denaturant systems, 0.7 M guanidine hydrochloride (GdnHCl) was added into each sample and the reaction was carried out exactly as described above. Melting curves were directly exported from the instrument, and then were analyzed with Prism 6 (GraphPad Software Inc.).

### SYPRO DSF assay: data analysis

DSF data were analyzed in three steps. First, raw fluorescence data as a function of temperature were fit to a modified form of the thermodynamic equation, as follows:

22 $$\Delta G=\Delta H(1-\frac{T}{{T}_{m}})-\Delta {C}_{p}({T}_{m}-T+T\,\mathrm{ln}[\frac{T}{{T}_{m}}])$$

23 $${K}_{U}={e}^{-\frac{\Delta G}{RT}}$$

24 $$Y(T)=[\frac{1}{1+{K}_{U}}({m}_{F}T+{b}_{F})]+[\frac{{K}_{U}}{1+{K}_{U}}({m}_{U}T+{b}_{U})]$$

Here Equation 24 relates the observed fluorescence signal, Y, as a function of temperature (T). The two terms in this equation correspond to contributions from folded and unfolded protein, respectively. Each term consists of the fraction of folded/unfolded protein, with a term that depends linearly on temperature (due to the temperature-dependence of the dye); thus, m_F_ and b_F_ capture this dependence of the dye when the protein is folded, as observed in the baselines before the thermal unfolded transition. The fraction of folded/unfolded protein at a given temperature depends on the “effective” unfolding/folding equilibrium constant (K_U_), which is dependent on both the temperature and the ligand concentration. As noted earlier, interaction between the dye and the protein is not explicitly included in this model of unfolding.

Throughout all of the analysis presented here, the value of ΔCp is held fixed. Thus, an individual thermal unfolding curve is fit using six free parameters: T_m_, ΔH, m_F_, b_F_, m_U_, and b_U_. Because the temperature dependence of the dye is the same regardless of the ligand concentration, however, we found that the fitting was be improved by using a single shared global parameter for the slopes of the baselines (m_F_ and m_U_).

From these fits, the fraction of unfolded protein can be determined at any temperature: collecting together data collected at different ligand concentrations for a single temperature of interest thus allows construction of the “isothermal” plots presented above.

To facilitate adoption of this approach, software is provided that carries out all of the analysis described herein. The software, and its associated user guide, is freely available for download via SourceForge (https://sourceforge.net/projects/dsf-fitting).

### Intrinsic Trp fluorescence

MBP was used at a final concentration of 2 µM in the buffer: 120 mM NaCl, 20 mM NaH_2_PO_4_/Na_2_HPO_4_, pH 7.4 and 1% DMSO. Data were collected with sample size 800 µL, in triplicate. All experiments were carried out using Photon Technology International (PTi) spectrophotometer with 4 × 10 mm quartz cuvettes. The excitation wavelength was set to 290 nm with 1 nm light pass-width and the emission wavelength was set to 337 nm (where Trp has the highest fluorescence intensity), with 6 nm light pass-width. The sample was pre-incubated for 10 minutes prior to measurement for each different temperature, from 25 °C to 72 °C. Fluorescence was measured continuously for 60 seconds at every temperature, and intensity values were averaged over this interval. The average fluorescence intensity over this interval was plotted as a function of temperature to obtain the thermal unfolding curve. Based on this unfolding curve, the T_m_ was estimated to be 56 °C.

This temperature was then used to measure fluorescence as a function of maltose concentration. Serial dilutions of maltose were prepared with 2 µM MBP (in the same buffer described above), and fluorescence was determined as described above. Data were analyzed via the same isothermal approach used for DSF data.

### Esterase activity assay

Carbonic anhydrase activity (*h*-CA I and *b*-CA II) was measured using a spectrophotometer (Molecular Devices, SpectraMax® i3x) as described elsewhere^73^. *h*-CA I was used at a final concentration of 2 µM and *b*-CA II was used at a final concentration of 0.5 µM in the buffer: 100 mM NaCl, 20 mM TRIS, pH 6.1, 1% DMSO. The substrate, 4-nitrophenyl acetate, was titrated through 0 to 3 mM from a freshly-prepared 3.2 mM stock. All reactions of *h*-CA I took place at 57 °C and reactions of *b*-CA II took place at 60 °C. The change in absorbance was measured at 348 nm. Enzyme initial velocity was plotted with different substrate concentration using Prism6. Data were collected for each of the four inhibitors, and the change in initial velocities were analyzed with the “Enzyme-noncompetitive inhibition” equation in Prism6.

## Supplementary information

Supporting Materials
