# Supplementary material for: Isothermal Analysis of ThermoFluor Data can readily provide Quantitative Binding Affinities
Source: Sci Rep. 2019 Feb 25;9:2650. doi: 10.1038/s41598-018-37072-x (PMC6389909; doi:10.1038/s41598-018-37072-x)
Supplement: Supplementary file 1 — Supporting Materials [file 41598_2018_37072_MOESM1_ESM.pdf]

Isothermal Analysis of ThermoFluor Data can readily provide Quantitative Binding Affinities

(Supplemental Materials)

Nan Bai[1,2], Heinrich Roder[1], Alex Dickson[3], and John Karanicolas[1*]

[1]Program in Molecular Therapeutics, Fox Chase Cancer Center, Philadelphia, PA 19111

[2]Department of Molecular Biosciences, University of Kansas, Lawrence, KS 66045

[3]Department of Biochemistry & Molecular Biology and Department of Computational Mathematics, Science and Engineering, Michigan State University, East Lansing, MI 48824

*To whom correspondence should be addressed. E-mail: **john.karanicolas@fccc.edu**, 215-728-7067

Supplemental Figures

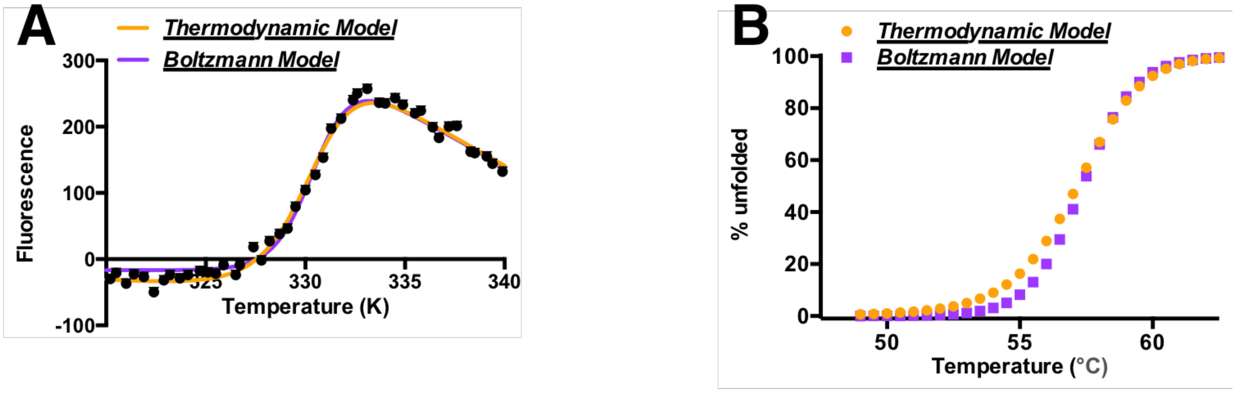

Supplemental Figure S1: The fraction of protein unfolding, as calculated from raw fluorescence data using various models to fit melting curve. (A) For analysis using both the thermodynamic model and the Boltzmann model, we included in the fitting linear (non-flat) baselines which necessary due to the temperature dependence of the dye. Both models fit the data well, but they differ slightly at the lower baseline. **(B)** These fits can be used to calculate the fraction of unfolded protein at different temperatures: results match closely between the two models, but they do exhibit a slight difference just below the transition region.

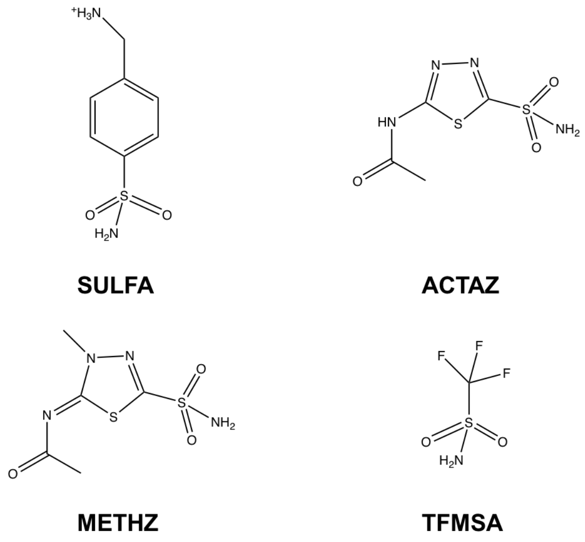

Supplemental Figure S2: Chemical structures of the carbonic anhydrase inhibitors used in this study.

Appendix

Detailed derivation of ***Equation 21***

We start from the basic equilibrium between protein folding-unfolding and protein-ligand binding presented in the main text:

$$[U] + [L] \underset{\mathrm{K_U}}{\rightleftharpoons} [F] + [L] \underset{\mathrm{K}_d}{\rightleftharpoons} [FL]$$

From here, we will consider the two specific conditions.

1) when no ligand is present:

$$[P]_T = [F]_0 + [U]_0 \tag{A.1}$$

$$[L]_T = [L]_0 = [FL]_0 = 0 \tag{A.2}$$

$$K_U = [U]_0/[F]_0 \tag{A.3}$$

$$f_{u0} = \frac{[U]_0}{[U]_0 + [F]_0} = \frac{1}{1 + 1/K_U} \tag{A.4}$$

Here, f_{u0} is the fraction unfolded protein in the absence of ligand.

Combining **Equation A.1** and **A.4** gives:

$$[U]_0 = f_{u0} \times [P]_T \tag{A.5}$$

$$K_U = \frac{f_{u0}}{1 - f_{u0}} \tag{A.6}$$

2) at the EC_{50}*:*

The EC_{50} is defined as the ligand concentration at which the initial fraction unfolded (f_{u0}) is reduced to half its original value. So:

$$[P]_T = [F]_{50} + [U]_{50} + [FL]_{50} \tag{A.7}$$

$$[L]_T = [L]_{50} + [FL]_{50} = EC_{50} \tag{A.8}$$

$$K_U = [U]_{50}/[F]_{50} \tag{A.9}$$

$$K_d = ([F]_{50} \times [L]_{50})/[FL]_{50} \tag{A.10}$$

$$[U]_{50} = \frac{[U]_0}{2} \tag{A.11}$$

where **Equation A.11** is derived from the definition of the EC_{50} (the fraction unfolded is half its original value). Combining **Equations A.9** and **A.11** gives:

$$[F]_{50} = \frac{[U]_{50}}{K_U} = \frac{[U]_0}{2\,K_U} \tag{A.12}$$

Combining **Equations A.7**, **A.11** and **A.12** gives:

$$[FL]_{50} = [P]_\mathrm{T} - [U]_{50} - [F]_{50} = [P]_\mathrm{T} - \frac{[U]_0}{2} - \frac{[U]_0}{2K_U} = [P]_\mathrm{T} - \frac{[U]_0}{2}\left(1 + \frac{1}{K_U}\right) \tag{A.13}$$

Combining **Equations A.5**, **A.6** and **A.13** gives:

$$[FL]_{50} = [P]_\mathrm{T} - \frac{[U]_0}{2} \times \left(1 + \frac{1 - f_{u0}}{f_{u0}}\right) = [P]_\mathrm{T} - \frac{[U]_0}{2} \times \frac{1}{f_{u0}} = [P]_\mathrm{T} - \frac{[P]_\mathrm{T}}{2} = \frac{[P]_\mathrm{T}}{2} \tag{A.14}$$

Combining **Equations A.5**, **A.6, A.10, A.12** and **A.14** gives:

$$[L]_{50} = [FL]_{50} \times \frac{K_d}{[F]_{50}} = \frac{[P]_\mathrm{T}}{2} \times K_d \times \frac{1}{\frac{[U]_0}{2K_U}} = \frac{[P]_\mathrm{T}}{2} \times K_d \times \frac{2K_U}{[U]_0}$$

$$= \frac{[P]_\mathrm{T}}{2} \times K_d \times \frac{\frac{f_{u0}}{1 - f_{u0}}}{\frac{1}{2}\,f_{u0}\,[P]_T} = \frac{[P]_\mathrm{T}}{2} \times K_d \times \frac{1}{\frac{1}{2}[P]_T(1 - f_{u0})}$$

$$= \frac{K_d}{1 - f_{u0}} \tag{A.15}$$

Combining **Equations A.8**, **A.14** and **A.15** gives:

$$EC_{50} = [L]_{50} + [FL]_{50} = \frac{K_d}{1 - f_{\mathrm{u}0}} + \frac{[P]_T}{2} \tag{A.16}$$

$$K_d = (1 - f_{\mathrm{u}0}) \times \left(EC_{50} - \frac{[P]_T}{2}\right) \tag{A.17}$$

The latter two equations (**A.16** and **A.17**) correspond to **Equations 20** and **21** in the main text.
